# Time to Follow-Up Colonoscopy After Positive Fecal Immunochemical Test with Centralized Patient Navigation: A Randomized Clinical Trial

**DOI:** 10.1007/s11606-025-10151-2

**Published:** 2026-02-04

**Authors:** Anisha P. Ganguly, Meghan C. O’Leary, Seth D. Crockett, Renée M. Ferrari, Connor M. Randolph, Lindsay R. Stradtman, Alexis A. Moore, Kevin Su, Xianming Tan, Alison T. Brenner, Daniel S. Reuland

**Affiliations:** 1 Lineberger Comprehensive Cancer Center, University of North Carolina at Chapel Hill, Chapel Hill, NC USA; 2 Division of General Medicine and Clinical Epidemiology, Department of Medicine, University of North Carolina at Chapel Hill, Chapel Hill, NC USA; 3 Department of Health Policy and Management, Gillings School of Global Public Health, University of North Carolina at Chapel Hill, Chapel Hill, NC USA; 4 Division of Gastroenterology and Hepatology, Department of Medicine, Oregon Health & Science University, Portland, OR USA; 5 Department of Maternal and Child Health, Gillings School of Global Public Health, University of North Carolina at Chapel Hill, Chapel Hill, NC USA; 6 Department of Health Behavior, Gillings School of Global Public Health, University of North Carolina at Chapel Hill, Chapel Hill, NC USA; 7 Department of Biostatistics, Gillings School of Global Public Health, University of North Carolina at Chapel Hill, Chapel Hill, NC USA; 8 Center for Health Promotion and Disease Prevention, University of North Carolina at Chapel Hill, Chapel Hill, NC USA

**Keywords:** patient navigation, safety-net providers, Survival analysis, colorectal neoplasms

## Abstract

**Background:**

Patients in federally qualified health centers (FQHCs) are at risk of delay in or non-completion of follow-up colonoscopy (FC) after a positive fecal immunochemical test (FIT). Increased time to FC is associated with increased colorectal cancer (CRC) incidence, late-stage diagnosis, and mortality.

**Objective:**

We evaluated the impact of centralized patient navigation on completion of FC and time to FC after a positive FIT.

**Design:**

This survival analysis is a sub-analysis of a randomized clinical trial conducted in FQHC systems in North Carolina. Trial patients were randomly assigned to mailed FIT outreach and to centralized patient navigation for a positive FIT or to usual care alone.

**Participants:**

RCT participants with a positive FIT.

**Intervention:**

Intervention patients with a positive FIT were offered centralized telephone-based navigation to FC, including support with procedure scheduling, bowel preparation, and social needs. Patients in the control arm received usual care.

**Main Measures:**

We compared the restricted mean time to FC in an intention-to-screen survival analysis over 1 year of follow-up. We censored by last observation date when FC was not completed.

**Key Results:**

Among 4002 trial patients, 842 completed a FIT, of whom 89 (10.6%) tested positive and were included in this analysis. Forty-eight (53.9%) were female, 29 (32.6%) identified as Black, 53 (59.6%) identified as White, and 53 (59.6%) had no prior CRC screening. Fifty-eight (65.2%) were intervention patients, and 31 (34.8%) received usual care (control). Intervention patients were more likely to complete FC at 1 year than control patients (69.0% vs 38.7%, *p* = 0.006). The difference in mean time to FC between the arms was 80.4 days (95% CI 13.6–147.2, *p* = 0.018). As-screened sensitivity analyses showed that the difference in time to FC increased further with increasing levels of engagement with navigation.

**Conclusions:**

Centralized patient navigation significantly increased FC completion and reduced the mean time to FC after a positive FIT among FQHC patients. Patient navigation is an important intervention to support the timely diagnostic resolution of positive CRC screening in under-resourced settings.

**Trial Registration:**

ClinicalTrials.gov Identifier: NCT04406714.

**Supplementary Information:**

The online version contains supplementary material available at 10.1007/s11606-025-10151-2.

## INTRODUCTION

Despite longstanding US Preventive Services Task Force recommendations for colorectal cancer (CRC) screening^1^, uninsured and underinsured, low-income, and racial/ethnic minority populations remain under-screened^2,3^. A key strategy to reach populations under-screened for CRC is the use of stool-based tests, such as the fecal immunochemical test (FIT), an effective, low-barrier, and low-cost self-testing modality^4,5^. Importantly, positive FITs require follow-up colonoscopy (FC) for diagnostic resolution and reduced risk of advanced colorectal neoplasia^6–12^. Yet, loss to follow-up between positive FIT and FC ranges from 16 to 38% in commercial integrated health systems^13^.

These challenges in CRC screening and FC completion are amplified in federally qualified health centers (FQHCs). A recent cross-sectional analysis of more than 16 million patients in 1364 FQHCs found a significant disparity in CRC screening completion (40.2% versus 72.3% in the US general population)^14^. To address this screening gap amidst cost and resource limitations, many FQHCs preferentially utilize FITs as a primary strategy for CRC screening over screening colonoscopy. However, delays and attrition between positive FIT and FC are exacerbated in the FQHC setting^15,16^. Systems factors including reliance on referrals external to FQHC systems, limited endoscopy availability in FQHC service areas, and care fragmentation contribute to delays and attrition of FQHC patients after positive FIT^15,17,18^. Moreover, FQHC patients are at increased risk for patient-level barriers to FC, including procedural concerns, transportation barriers, insufficient coverage and out-of-pocket costs, and mistrust^19–21^.

Prior research has demonstrated the effectiveness of patient navigation and population health-based outreach for stool-based testing in increasing CRC screening uptake^22–25^, particularly through multicomponent interventions^26^. Patient navigation is considered an evidence-based practice for increasing cancer screening among individuals from lower income and historically disadvantaged racial/ethnic populations^27^. Previous research has established the effectiveness of patient navigation to ensure FC completion after a positive FIT^26,28^however, the effect of patient navigation on reducing time to FC, a critical factor in reducing the risk of CRC incidence and mortality^6,8^, has not been well characterized. Furthermore, exemplary models of patient navigation after a positive FIT are largely within integrated health systems^24,29,30^. A recent study by Coronado and colleagues among patients with a positive FIT in a Washington state FQHC system showed that patient navigation increased FC completion by 13% and reduced mean time to FC by 27 days^31^. Additional studies across healthcare contexts, including the fragmented FQHC setting, and with contrasting implementation strategies for navigation, are needed to further validate the clinical impact of patient navigation for FC after a positive FIT.

We recently showed that a multicomponent intervention comprised of centralized mailed FIT outreach and navigation for patients with positive FITs among FQHC patients increased both CRC screening and the rate of detection of advanced colorectal neoplasia compared with usual care alone^32^. In this analysis, we sought to evaluate the impact of the centralized patient navigation component of the intervention on reducing time to FC among patients with a positive FIT compared with usual care. We hypothesized that patients randomized to receive the intervention would have reduced time to FC due to the benefits of centralized patient navigation.

## METHODS

In this sub-analysis, we compared time to FC among patients with positive FITs across study arms of a larger pragmatic randomized clinical trial (NCT04406714) using an intention-to-screen Kaplan-Meier survival analysis. This study followed the Consolidated Standards of Reporting Trials (CONSORT) reporting guideline and was approved by the Institutional Review Board at the University of North Carolina at Chapel Hill. The randomized trial was comprised of 4002 patients receiving care in two FQHC systems in North Carolina. Eligible patients were randomized 1:1 using permuted blocks with varying sizes per wave, stratified by insurance type and FQHC site. Centralized mailed FIT outreach was associated with a 20.3-percentage point increase (30.0% vs. 9.7%, 95% CI: 17.9–22.7) in primary CRC screening uptake within 6 months compared to usual care alone^32^. Here, we assessed the effect of the centralized patient navigation component of the intervention on time to FC among patients with positive FITs.

### Patient Navigation

Intervention patients were eligible to receive centralized patient navigation after a positive FIT to support them in completing an FC alongside usual care. As previously described^32–34^, the centralized outreach team included a full-time, bilingual patient navigator (CMR) with an undergraduate degree and 2 years of experience in care coordination for uninsured populations in North Carolina. The navigator was not clinically licensed nor employed by either FQHC system. Rather, the navigator was employed by the study team, was physically located at the academic center more than 150 miles away from the clinical sites, and delivered remote, telephone-based navigation services with electronic health record (EHR) access for both FQHC systems. The navigation protocol was adapted from the New Hampshire patient navigation program^35,36^, and generally included CRC screening education, colonoscopy scheduling assistance, barriers assessment and resolution, bowel prep education, and post-procedure follow-up to review results and future screening/surveillance recommendations.^37^The patient navigator was formally trained in motivational interviewing^38^, an evidence-based approach to identify possible barriers, elicit individual assets and capabilities, and develop self-efficacy to complete screening. All patient interactions with patients were documented using Research Electronic Data Capture (REDCap) navigation call logs. To support the holistic assessment of social needs, fields were included for specific types of barriers—namely, transportation, financial, emotional, informational, and other barriers to colonoscopy. The patient navigator arranged services for health-related social needs such as transportation assistance and addressed patient-specific questions.

The patient navigator attempted to contact intervention patients with positive FITs up to three times by phone after they had been notified about their result by their clinical team. The time from a positive FIT result to initial navigator contact was a median of 9 days (range: 1–77 days). Patients who could not be reached by phone were mailed a letter about the importance of FC and how to receive navigation support. Among patients who received navigation, the median number of navigation calls was 5 (range: 1–20 calls) (unpublished data).

Patients in the control arm received only usual care and were not offered centralized patient navigation. In usual care, an FQHC provider or clinical staff member notified patients about their positive FIT, and the provider generated a referral to GI. Based on clinic feedback, there was variation within and across clinics in how missed appointments or scheduling failures were handled. However, beyond having a referral coordinator who assisted with the initial colonoscopy referral, there were no systematic navigation services provided to patients with a positive FIT at either FQHC.

### Study Sample

We included all trial patients who had a confirmed positive FIT in the intervention or the control arm based on chart review during the first round of mailed FIT outreach (per the study protocol, the intervention patients were eligible for a second round of screening 1 year after the first round of FITs was mailed)^32,39^. Randomization for the first round of outreach occurred in waves between July 2020 and September 2021.

### Outcomes

A chart review was performed between February 2022 and October 2023. FC completion and endoscopic pathology results were assessed through a dual manual review of EHR data, with resolution provided by a blinded clinician investigator for discrepancies. We determined the total observation time for up to 1 year for each patient with a positive FIT based on an observation start date from when the positive FIT result was released in the EHR. If an FC was completed, the observation period ended on the FC date. If there was no evidence that an FC was performed, we censored the observation time on the most recent date of observation (i.e., the latest chart review date) up to 1 year of follow-up.

### Intention-to-Screen Analysis

As our primary endpoint analysis, we conducted an intention-to-screen analysis and assessed whether time to FC differed between patients with a positive FIT who were randomized to the intervention versus the control.

### Sensitivity Analyses

We conducted three as-screened sensitivity analyses to evaluate potential differences in time to FC based on increasing levels of engagement with patient navigation. In the first, we compared time to FC between patients who specifically completed a mailed FIT through the trial versus all remaining patients (from both the intervention and control arms) who completed a FIT through usual care. We included this analysis because some intervention patients completed FITs during usual care (rather than through the mailed study FIT), and these individuals were not offered centralized patient navigation. The other two sensitivity analyses focused on levels of engagement with patient navigation, as determined through the navigator call logs. We defined navigation engagement consistently with our prior analyses^32,40^. Patients were “reached” if the navigator was able to successfully contact them by phone to offer navigation services. Patients “received” navigation if they participated in at least one navigation call. We compared time to FC between patients who were successfully reached by the patient navigator versus control patients and all other intervention patients who were not successfully contacted. Additionally, we compared time to FC between those who received patient navigation versus all other intervention patients (i.e., those who declined navigation or were not reached) and control patients.

### Statistical Analysis

Baseline characteristics, observation time, source of the completed FIT, colonoscopy completion at 12 months, and endoscopic results were compared using chi-squared tests and t-tests. We utilized the Kaplan-Meier method to generate survival curves and compare time to FC in an intention-to-screen analysis as well as in sensitivity analyses. Given the high rate of censoring, differentially in the control arm, we utilized restricted mean survival time^40^ over 12 months to compare restricted mean time to FC and to calculate the difference in time to FC across arms. All analyses were conducted in Stata 18 (Copyright StataCorp LLC, College Station, TX).

## RESULTS

**Figure 1:**
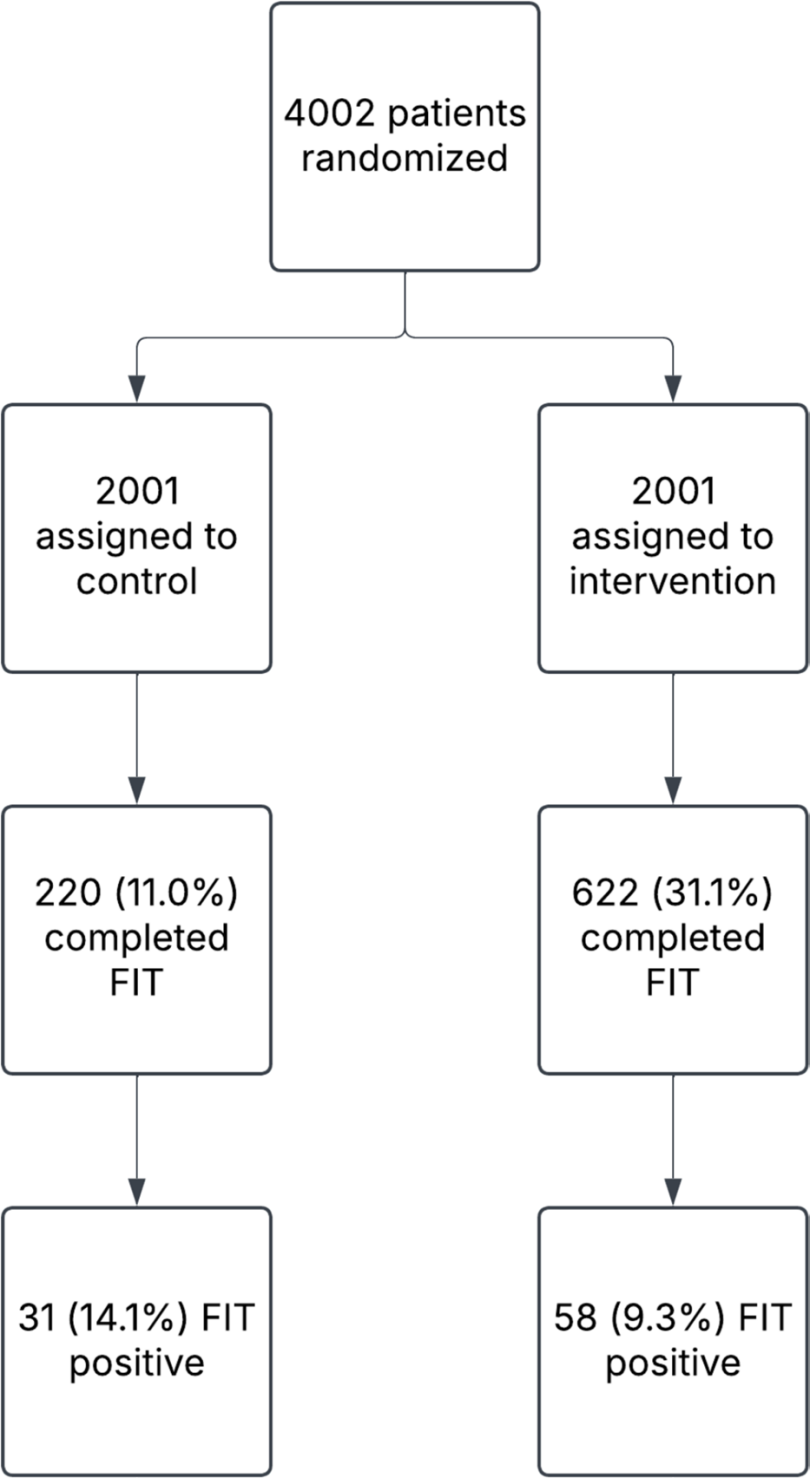
**Study flow diagram of patients with **a **positive fecal immunochemical test by trial arm.**

Among 4002 patients randomized (Fig. 1), 842 (21.0%) completed a FIT, of whom 622 (73.9%) were in the intervention arm and 220 (26.1%) were in the control arm. Eighty-nine (10.6%) patients who completed a FIT tested positive: 58 (65.2%) were intervention patients and 31 (34.8%) were control patients (Table 1). The mean age of patients with a positive FIT was 60.8 years, and 53.9% were female. Patients with a positive FIT were comprised of 59.6% non-Hispanic White and 32.6% non-Hispanic Black individuals. Most patients’ preferred language was English (80.9%). Patients’ primary insurance included Medicare (32.6%), Medicaid (18.0%), commercial (22.5%), and self-pay/uninsured (27.0%). Most patients (59.6%) had no EHR evidence of prior CRC screening. There were no significant differences in age, sex, race/ethnicity, preferred language, primary insurance, or screening history by arm among patients with a positive FIT. Observation time was similar across arms (mean 156.7 days [95% CI 112.1–201.4] vs 192.6 days [95% CI 133.8–251.4], *p* = 0.34). Among intervention patients, 43 (74.1%) completed a FIT through the intervention mail distribution, while 15 (25.9%) completed a FIT distributed by their clinic.

**Table 1:** Baseline Characteristics of Federally Qualified Health Center Patients with Positive Fecal Immunochemical Test (FIT) by Trial Arm

	Total *N* = 89 (100.0%)	Intervention *N* = 58 (65.2%)	Control *N* = 31 (34.8%)	*P*-value
Age group				
50–54	23 (25.8%)	17 (29.3%)	6 (19.4%)	0.06
55–59	20 (22.5%)	9 (15.5%)	11 (35.5%)	
60–64	19 (21.3%)	10 (17.2%)	9 (29.0%)	
65–69	13 (14.6%)	11 (19.0%)	2 (6.5%)	
70–74	14 (15.7%)	11 (19.0%)	3 (9.7%)	
Sex				
Male	41 (46.1%)	28 (48.3%)	13 (41.9%)	0.57
Female	48 (53.9%)	30 (51.7%)	18 (58.1%)	
Race/Ethnicity				
Non-Hispanic White	53 (59.6%)	36 (62.1%)	17 (54.8%)	0.42
Non-Hispanic Black	29 (32.6%)	19 (32.8%)	10 (32.3%)	
Other*	7 (7.9%)	3 (5.2%)	4 (12.9%)	
Preferred language				
English	72 (80.9%)	45 (77.6%)	27 (87.1%)	0.42
Spanish	2 (2.2%)	2 (3.4%)	0 (0.0%)	
Unknown	15 (16.9%)	11 (19.0%)	4 (12.9%)	
Primary insurance				
Medicare	29 (32.6%)	23 (39.7%)	6 (19.4%)	0.16
Medicaid	16 (18.0%)	11 (19.0%)	5 (16.1%)	
Commercial	20 (22.5%)	10 (17.2%)	10 (32.3%)	
Self-pay/Uninsured	24 (27.0%)	14 (24.1%)	10 (32.3%)	
CRC screening history				0.84
Previously screened	36 (40.4%)	23 (39.7%)	13 (41.9%)	
Never been screened	53 (59.6%)	35 (60.3%)	18 (58.1%)	
Mean observation time, in days (95% CI)	169.2 (134.1–20.4.3)	156.7 (112.1–201.4)	192.6 (133.8–251.4)	0.34
Source of completed, positive FIT				
By mail	43 (48.3%)	43 (74.1%)	–	
Distributed by clinic	46 (51.7%)	15 (25.9%)	31 (100.0%)	
Colonoscopy completion within 1 year of positive FIT	52 (58.4%)	40 (69.0%)	12 (38.7%)	0.006

^*^Other race/ethnicity includes Hispanic (*n* = 3), Asian/Native Hawaiian or Pacific Islander (*n* = 2), and unknown (*n* = 2)

**Figure 2:**
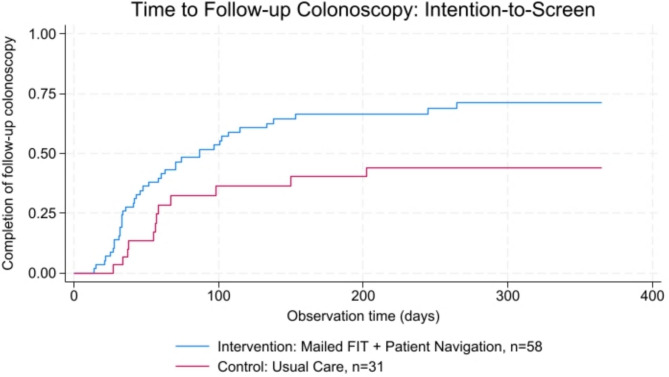
**Intention-to-screen Kaplan-Meier estimate of time to follow-up colonoscopy among patients with **a **positive fecal immunochemical test (FIT), *n* = 89.**

In the intention-to-screen analysis (Fig. 2), 69.0% of intervention patients completed FC up to 12 months after positive FIT compared to 38.7% of control patients (*p* = 0.006). Restricted mean time to FC was 157.0 days (95% CI 120.0–194.0) for intervention patients and 237.4 days (95% CI 181.9–293.0) for control patients, a difference of 80.4 days (95% CI 13.6–147.1, *p* = 0.018).

**Figure 3:**
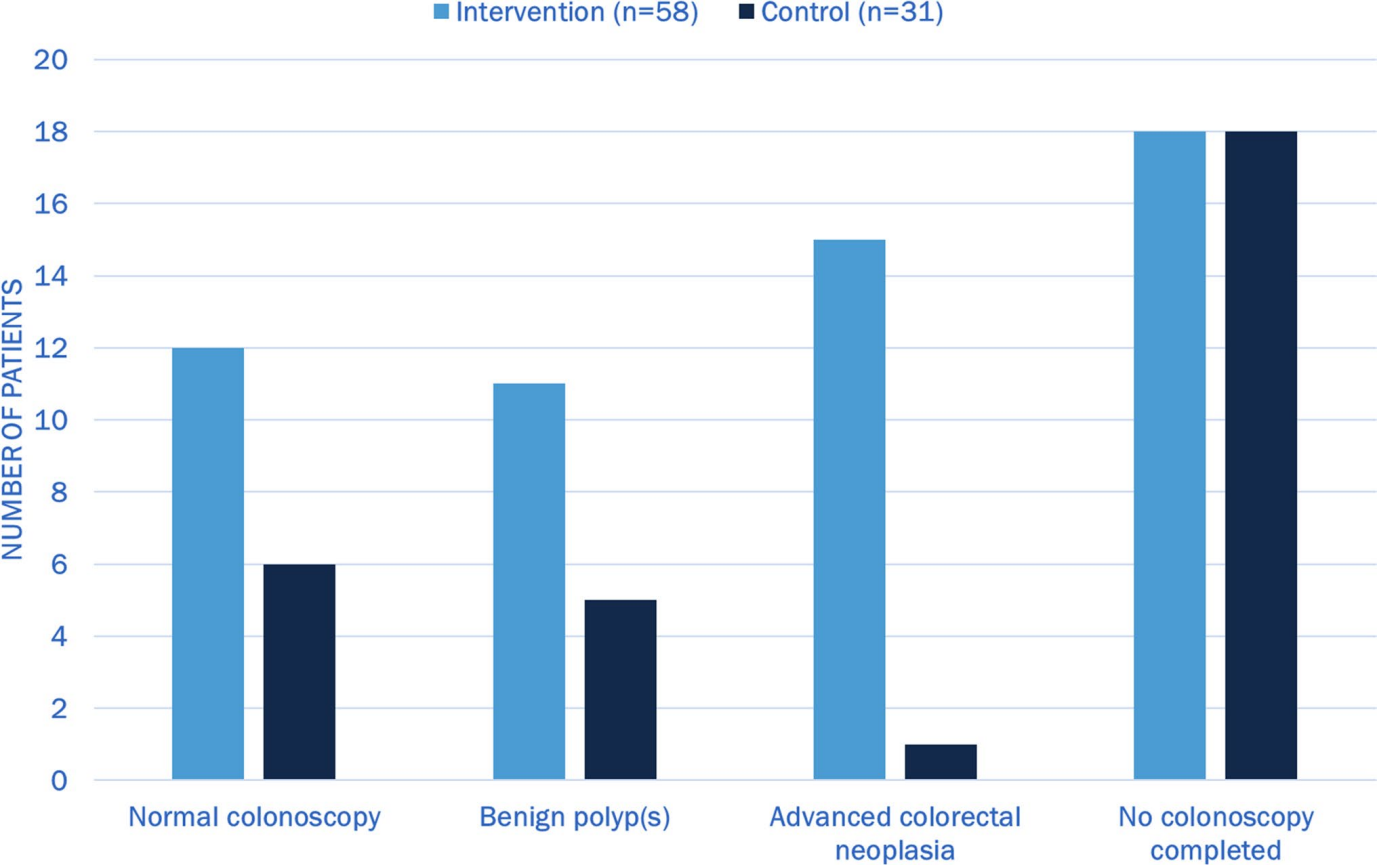
Endoscopic results of patients with a positive fecal immunochemical test (FIT)*. *Endoscopy pathology results were missing for 2 patients in the intervention arm and 1 in the control arm.

Endoscopic pathology results were available for 86 of 89 patients with a positive FIT (Fig. 3). Among intervention patients, 15 (26.8%) were found to have advanced colorectal neoplasia, compared with 1 (3.3%) control patient (*p* = 0.02).

**Figure 4:**
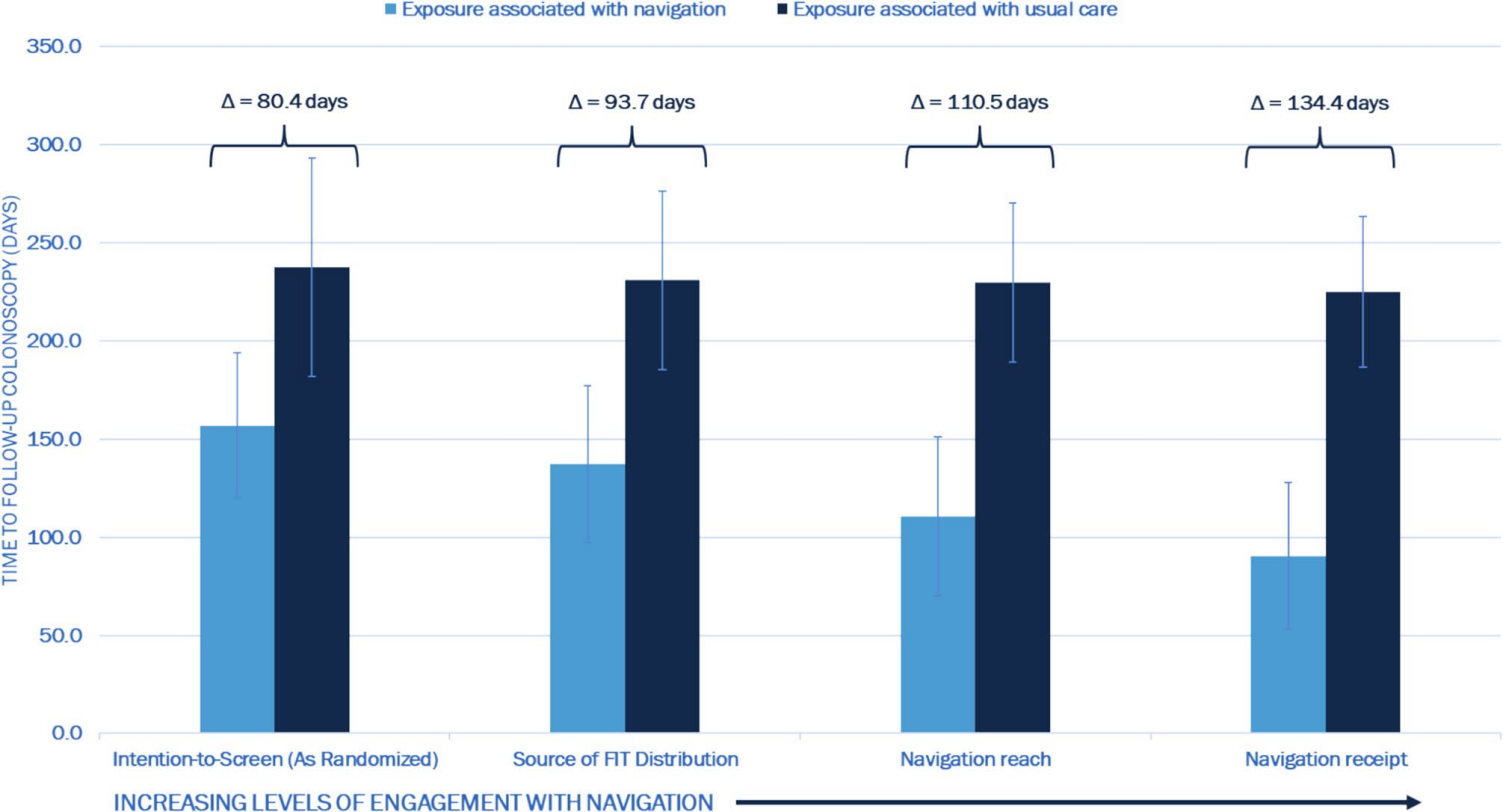
As-screened sensitivity analyses of time to follow-up colonoscopy by increasing levels of engagement with patient navigation, n=89. *Intention-to-screen compared participants randomized to the **intervention (*n* = 58) vs. **the **control (*n* = 31), (*p* = 0.02); mode of fecal immunochemical test (FIT) distribution compared **the **intervention mailed FIT (*n* = 43) vs. FIT distributed through clinical care (*n* = 46), (*p* = 0.002); navigation reach compared patients reached by the navigator (*n* = 33) vs. patients not reached, including **the **control arm (*n* = 56), (*p* < 0.001); and navigation receipt compared patients accepting and receiving navigation (*n* = 26) vs. patients who declined or were not offered navigation (*n* = 63), (*p* < 0.001).**

**Figure 5:**
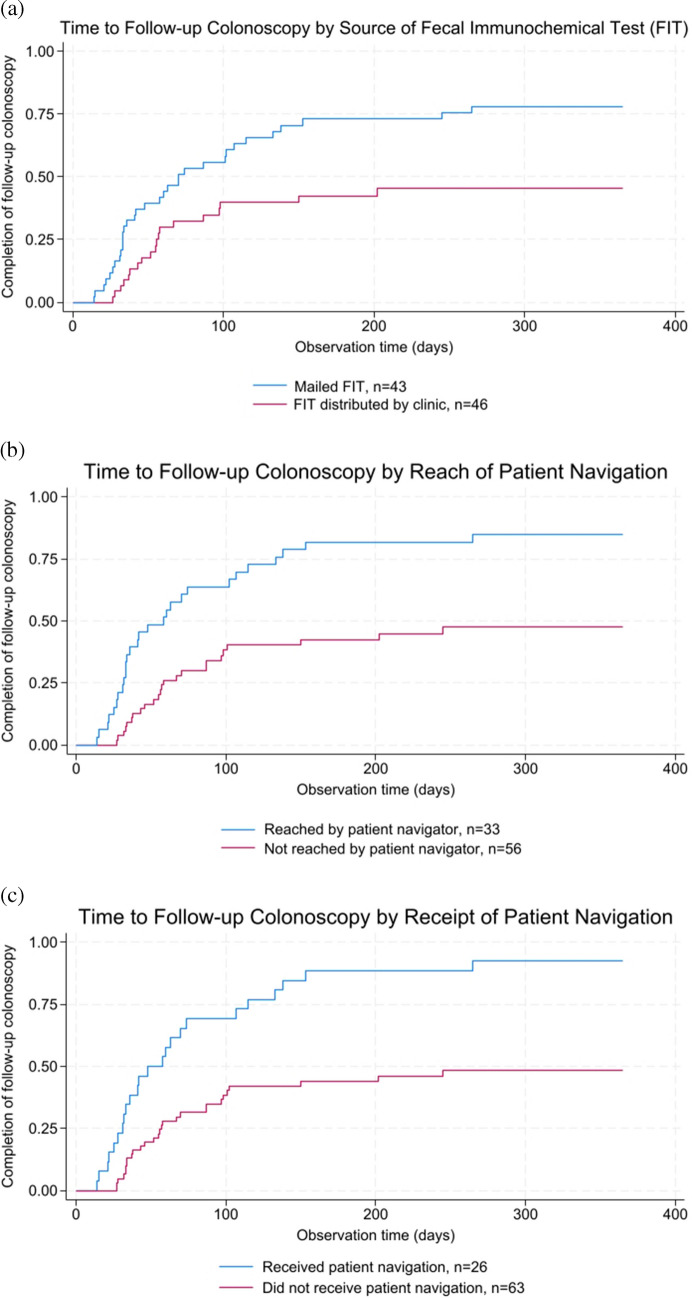
Sensitivity analyses exploring Kaplan-Meier estimate of time to follow-up colonoscopy among patients with positive FIT by increasing levels of engagement with patient navigation

An as-screened sensitivity analysis comparing the source of FIT distribution (intervention mailed FIT versus FIT through usual care) showed a similar reduction in time to FC (Figs. 4 and 5a), with a restricted mean of 137.2 days (95% CI 97.2–177.2) for mailed FITs and 230.9 days (95% CI 185.3–276.4) for clinic-based FITs, a difference of 93.7 days (95% CI 33.1–154.3, *p* = 0.002).

 Figures 4, 5b and c present the effect of increasing levels of navigation on time to FC among patients with a positive FIT. Among the 58 patients randomized to the intervention arm, 33 (37.1% of patients with a positive FIT) were successfully reached by the navigator, of whom 26 (29.2%) ultimately received navigation. Patients reached by the navigator had a restricted mean time to FC of 110.5 days (95% CI 69.9–151.0), while patients not reached had 229.9 days (95% CI 189.3–270.4), a difference of 119.4 days (95% CI 62.1–176.8, *p* < 0.001). Patients receiving navigation had an even shorter time to FC: a restricted mean of 90.5 days (95% CI 53.4–127.7) compared to 225.0 days (95% CI 186.5–263.4) among those who did not receive navigation, a difference of 134.4 days (95% CI 81.0–187.9, *p* < 0.001).

## DISCUSSION

In this clinical trial, patients receiving centralized patient navigation after a positive FIT had nearly double the rate of colonoscopy completion and a reduction in time to FC of approximately 3 months. Sensitivity analyses showed further reduction in time to FC among participants who had more engagement with patient navigation, suggesting a “dose-response” effect that reinforces our findings.

In our study, the observed reduction in time to FC of nearly 3 months among intervention patients suggests a clinically significant difference in time to diagnostic resolution and a decreased risk of CRC incidence. A prior microsimulation model by Meester et al^41^. found an increased CRC incidence risk of 0.3%, and an increased CRC mortality risk of 1.4%, per month from positive screening test to FC, emphasizing the sizable clinical impact of the reduced time to FC in the intervention arm. Of note, previous analyses have demonstrated increased risk of CRC incidence and advanced-stage disease with 6–10 months delay in receipt of FC^8,11,42^; mean time to FC was reduced from 8 to 5 months in our study. Limited previous studies exploring time to FC and patient navigation in large, integrated health systems have shown reductions of 15 days in a randomized trial and 50 days in an observational study^29,30^; our findings suggest an even larger benefit in the FQHC population. The increased detection of advanced colorectal neoplasia in the intervention arm compared to the control arm (26.9% vs 3.3%) underscores the clinical significance of this reduction in time to FC, though this exploratory outcome is limited by sample size. In the parent trial, we showed that among all trial participants (*n*= 4002), detection of advanced colorectal neoplasia on colonoscopy completed for screening or follow-up was more than 1.5 times higher among intervention patients compared to patients randomized to the control arm^32^.

This study builds upon prior research demonstrating the effectiveness of centralized patient navigation across the cancer screening and diagnostic continuum. Over the past 30 years, patient navigation has been well-established as an intervention to improve screening uptake across cancer types^21,43^. A 2011 systematic review noted less evidence for patient navigation for diagnostic resolution after abnormal screening^44^; subsequent studies in Denver, CO^28^, Columbus, OH^45^, and Washington state^46^ showed a reduction in time to diagnostic resolution after abnormal breast, cervical, and CRC screening with patient navigation. These prior studies were conducted within integrated health systems and utilized patient navigators employed within the health systems where patients received care.

Until recently, robust models of patient navigation for diagnostic resolution after positive CRC screening tests were lacking in the FQHC setting. A recent trial conducted in a Western Washington FQHC system with 32 clinics showed that FC completion increased by 13 percentage points and that time to FC was reduced from 256 to 229 days among patients randomized to patient navigation^31^. Our study complements these findings by replicating the benefits of patient navigation for FC after positive FIT in another environment. We similarly observed an improvement in FC completion (increase of 30 percentage points) and a reduction in time to FC (from 237 to 157 days) in a distinct geographic setting and healthcare landscape—two FQHC systems in Western and Northeastern North Carolina, both rural areas, from 2020 to 2022, prior to state Medicaid Expansion. The increased effect sizes of FC completion and the difference in time to FC with patient navigation in our study may reflect the opportunity for even greater impact in highly uninsured and geographically isolated communities. Additionally, our study shows the effective implementation of a remote patient navigator, without clinical credentialing, centralized across FQHC systems. In contrast to the Washington FQHC trial^31^, where a total of 9 different clinic staff members served as the patient navigators due to personnel turnover, our study engaged a single remote navigator who delivered navigation services throughout the full 2-year study period. The implementation of a centralized navigator is appealing because of the opportunity to scale navigation without drawing upon strained FQHC resources or relying on healthcare personnel to deliver navigation services. Such centralized navigation strategies can be scaled through similar academic-community partnerships or even payer-based navigation interventions in the future.

Importantly, this analysis provides further insight into centralized patient navigation as an intervention for reducing cancer health disparities. Addressing disparate access to gastroenterology care for socially vulnerable populations requires effective engagement and patient-centered outreach, including health education to prepare for GI procedures and to address social barriers^47^. Our findings show that centralized navigation addressing those needs effectively ensured retention through diagnostic resolution among marginalized populations, including a notable proportion of screening-naïve patients (59.6%). In a prior meta-analysis of 22 trials evaluating the impact of patient navigation on cancer screening rates in populations experiencing health disparities^48^, patient navigation was associated with higher CRC screening overall, and especially by colonoscopy, compared to usual care. FQHCs, the setting for this work, are large safety-net providers for low-income, uninsured, Medicaid-insured, and racial/ethnic minority populations^49^. A recent analysis of patients’ self-reported social risk factors showed that more than 25% of FQHC patients screened positive for financial strain and more than 1 in 10 screened positive for food insecurity, housing insecurity, and transportation barriers, particularly among patients with lower income, without insurance, or self-identifying as Black^50^. Patients seeking care at FQHCs are demonstrably under-screened for guideline-recommended cancer screening^14^, and are even more so at risk for attrition after positive cancer screening due to health-related social needs^15,18^. Our findings affirm centralized patient navigation as a targeted intervention for FQHC patients experiencing health-related social needs that pose a barrier to CRC screening and diagnostic resolution.

Previous work studying the effect of patient navigation in chronic disease has suggested that the reach of patient navigation is a critical determinant of navigation effectiveness^51,52^. A review by Freund^53^ regarding the implementation of patient navigation programs in cancer care noted the importance of systems processes to identify and track patient receipt of navigation. In our study, centralized navigation, including a detailed process evaluation of navigation reach and receipt, allowed for an evaluation of navigation implementation and correlation of different levels of engagement with navigation to diagnostic resolution after a positive FIT. We found a quasi-dose responsive relationship between successful reach by the navigator and patient receipt of navigation and reduced time to FC among patients with a positive FIT, supporting the assertion that clinical effectiveness from centralized patient navigation is driven by the level of engagement with navigation.

Notably, this trial took place during peak conditions of the COVID-19 pandemic. Patient navigation for colonoscopy completion may have been even more impactful in this context given the increased complexity of navigating procedure scheduling amidst clinic closures, staffing challenges, pre-procedure COVID-19 testing, and transportation disruptions^54,55^. Importantly, the patient navigator in this study addressed questions such as COVID-19 testing requirements. These pandemic conditions may have amplified the differences in effect sizes between those randomized to patient navigation and those receiving usual care in a strained healthcare system. Nonetheless, the effectiveness of patient navigation amidst pandemic conditions highlights the value of centralized patient navigation in complex systems and external challenges.

These findings should be interpreted within the study’s limitations. As this sub-analysis derives from a larger trial testing the mailed FIT component of the intervention, the sample of patients with positive FITs had a greater percentage of intervention patients (65.2%) than control patients (34.8%), though there were no differences in baseline characteristics. It is possible that there were missing outcome data from patients who completed colonoscopies not reflected in this analysis due to incomplete procedure documentation and specialty care fragmentation^56,57^. However, we undertook extensive efforts to track colonoscopy referrals and obtain records from endoscopy providers for all positive FITs. Furthermore, because of randomization, it is unlikely that our results are explained by differential missingness across trial arms. Confidence intervals of point estimates for restricted mean survival time were wide, reflecting the limited sample size of patients with positive FITs; future studies should recruit patients with positive FITs to ensure larger sample sizes to improve precision. As this study was conducted among under-screened rural communities in North Carolina, these findings may not generalize to urban settings in other states. Additionally, this trial took place during the COVID-19 pandemic, which posed disruptions to clinical care, particularly non-emergent procedures like follow-up colonoscopies^54,55^; this may limit the generalizability of our findings.

## CONCLUSION

Using data from a pragmatic randomized clinical trial, we found that centralized patient navigation significantly reduced the time to FC and facilitated timely diagnostic resolution among FQHC patients with a positive FIT. Closer engagement with the navigator further reduced the time to FC. Our future work will evaluate the cost-effectiveness of centralized patient navigation after positive FITs, as well as the critical components of our patient navigation program to ensure its efficacy in different settings. These efforts can inform policy around payment for patient navigation and the adoption of similar models of care in new settings and contexts.

## Supplementary Information

Below is the link to the electronic supplementary material.

ESM 1 (PDF 409 KB)

ESM 2 (DOCX 50.3 KB)

ESM 3 (PNG 205 KB)

ESM 3 (TIF 409 KB)

## Data Availability

Deidentified participant data and a codebook are accessible to anyone requesting these data for any purpose by submitting a request through the ACCSIS Data Share website, https://healthcaredelivery.cancer.gov/accsis/datashare.
