# Supplementary material for: Time to Follow-Up Colonoscopy After Positive Fecal Immunochemical Test with Centralized Patient Navigation: A Randomized Clinical Trial
Source: J Gen Intern Med. 2026 Feb 4;41(6):1534–44. doi: 10.1007/s11606-025-10151-2 (PMC13007977; doi:10.1007/s11606-025-10151-2)
Supplement: Supplementary file 1 — (PDF 409 KB) [file 11606_2025_10151_MOESM1_ESM.pdf]

LCCC1826: Scaling Colorectal Cancer Screening Through Outreach, Referral, and Engagement (SCORE): A Program to Reduce Colorectal Cancer Burden in Vulnerable Populations

Principal Investigator
Daniel Reuland, MD, MPH
UNC Lineberger Comprehensive Cancer Center
101 East Weaver Street, Campus Box 7293
Carrboro, NC 27510
919-391-7775 (phone)
Email: daniel_reuland@med.unc.edu

Principal Co-Investigator
N/A

Co-Investigator(s)
Alison Brenner, PhD, MPH
Seth Crockett, MD, MPH
Leah Frerichs, PhD
Kristen Hassmiller Lich, PhD, MHSA
Jennifer Leeman, DrPH, MPH
Teri Malo, PhD
Shana Ratner, MD, FACP
Catherine Rohweder, DrPH
Karyn Stitzenberg, MD, MPH, FACS
Xianming Tan, PhD
Stephanie Wheeler, PhD, MPH

Biostatistician
Xianming Tan, PhD

Sponsor: Lineberger Comprehensive Cancer Center

Funding Source: Colon Cancer Coalition and National Cancer Institute

Version Date: v3 03/30/20

LCCC1826: Scaling Colorectal Cancer Screening Through Outreach, Referral, and Engagement (SCORE): A Program to Reduce Colorectal Cancer Burden in Vulnerable Populations

Principal Investigator
Daniel Reuland, MD, MPH
UNC Lineberger Comprehensive Cancer Center
101 East Weaver Street, Campus Box 7293
Carrboro, NC 27510
919-391-7775 (phone)
Email: daniel_reuland@med.unc.edu

Signature Page

The signature below constitutes the approval of this protocol and the attachments, and provides the necessary assurances that this trial will be conducted according to all stipulations of the protocol, including all statements regarding confidentiality, and according to local legal and regulatory requirements and applicable U.S. federal regulations and ICH guidelines.

Principal Investigator (PI) Name:__Daniel Reuland____________________

PI Signature: __________________

Date:___03/30/20________________

Version Date:__v3 03/30/20____________________

TABLE OF CONTENTS

1.0 BACKGROUND AND RATIONALE 1
1.1 Study Synopsis 1
1.2 Background 1
1.3 Purpose and Rationale 2
2.0 STUDY OBJECTIVES AND ENDPOINTS 3
2.1 Primary Objective (Aim 1) 3
2.2 Secondary Objectives 3
3.0 PATIENT ELIGIBILITY 3
3.1 Inclusion Criteria 3
3.2 Exclusion Criteria 4
4.0 STUDY PLAN 4
4.1 Schema 5
4.2 Duration of Study 7
4.3 Study Details 7
4.3.2 Randomization 7
4.4 Expected Risks 11
4.5 Removal of Patients from Protocol 11
5.0 TIME AND EVENTS TABLE 11
5.1 Time and Events Table 11
6.0 UNANTICIPATED PROBLEMS 12
6.1 Definition 12
6.2 Reporting 13

7.0 STATISTICAL CONSIDERATIONS 13

7.1 Study Design 13

7.2 Sample Size and Accrual 13

7.3 Data Analysis Plans 14

7.4 Data Management/Audit 16

8.0 STUDY MANAGEMENT 16

8.1 Institutional Review Board (IRB) Approval and Consent 16

8.2 Required Documentation 17

8.3 Registration Procedures 17

8.4 Adherence to the Protocol 17

8.5 Amendments to the Protocol 18

8.6 Record Retention 18

8.7 Obligations of Investigators 19

9.0 REFERENCES 19

1.0 BACKGROUND AND RATIONALE

1.1 Study Synopsis

This study assesses the effectiveness, feasibility, acceptability, and cost-effectiveness of mailed fecal immunochemical test (FIT) screening, compared to usual care, among patients served by Roanoke Chowan Community Health Center (RCCHC) and Blue Ridge Health (BRH) who are due for colorectal cancer screening (CRC) according to US Preventive Services Task Force guidelines for improving CRC screening rates. It includes formative work to develop and refine the intervention implementation and outcomes measurement, as well as a randomized controlled trial. We will work with RCCHC and BRH to identify up to 4,000 patients who are potentially eligible for CRC screening. Patients will be randomized to the following two arms: usual care (Arm 1) and mailed FIT plus up to two mailed reminders to complete the FIT (Arm 2). Current usual care at RCCHC and BRH consists of a visit-based FIT distribution approach. We hypothesize that we will observe higher CRC screening completion rates among patients assigned to study Arm 2. Our economic outcomes are the per-arm total costs and the incremental cost-effectiveness of intervention versus usual care arms, measured in cost per person screened for CRC.

1.2 Background

CRC is the third most common cancer diagnosis and the second leading cause of cancer death in the United States (U.S. Cancer Statistics Working Group, 2013). Screening is effective in reducing CRC incidence and mortality (United States Preventive Services Task Force et al., 2016), but CRC screening is underused. Although CRC screening has increased since 2002, current screening rates remain modest, with only 68% of age-eligible adults in North Carolina up-to-date with screening in 2012 (Centers for Disease Control and Prevention [CDC], 2013).

In North Carolina, as in many parts of the United States, screening is substantially underused in vulnerable and marginalized populations (CDC, 2013). CRC burden is particularly high in certain regions of the state. A recent study identified one such region ("hotspot") in northeastern North Carolina with elevated CRC mortality rates compared to national averages (Siegel et al., 2015). Hertford County, in which RCCHC is located, is one of 11 counties in North Carolina that is included in this "hotspot." This "hotspot" is a priority area for interventions to increase CRC screening. The western region of North Carolina, in which BRH is located, is a mountainous area characterized by rural, largely white, Appalachian communities as well as large and growing communities of Hispanic immigrants who work in the agricultural sector (e.g., the Christmas tree industry). Both regions afford a critical opportunity to address disparities in CRC screening and disease burden.

Increasing CRC screening among vulnerable populations will be crucial to reach the Healthy People 2020 and National Colon Cancer Roundtable goals of having

70.5-80% of the age-eligible population up-to-date with CRC screening (National Colorectal Cancer Roundtable, n.d.; Office of Disease Prevention and Health Promotion, 2014).

The United States Preventive Services Task Force (2016) recommends several stand-alone tests to screen for CRC, including colonoscopy every 10 years and fecal blood tests such as fecal immunochemical testing (FIT) annually, for patients ages 50-75 years. Further, the CDC Community Guide to Prevention recognizes several effective and cost-effective means of increasing CRC screening, including interventions to decrease structural barriers (such as mailed, at-home stool testing) (Sabatino et al., 2012). Mailed programs of FIT have shown promise as an effective means of increasing screening use, including for vulnerable populations. One program showed that a mailed FIT-based outreach program could increase screening by nearly 30 percentage points among vulnerable patients in a safety net system in Texas (Gupta et al., 2013). Several other studies have found mailed fecal testing programs to be effective in increasing screening rates in insured populations (Church et al., 2004; Kempe et al., 2012; Levy et al., 2012); however, to date, such programs have not been implemented and tested in North Carolina’s vulnerable populations.

Through this study, we will work with federally qualified health centers (FQHCs; RCCHC and BRH) to help increase the rate of CRC screening completion among their screening-eligible patients, including underserved adults. Approximately 54% of screening-eligible patients at RCCHC are up-to-date with CRC screening, leaving about 46% (*n* = ~3,100) patients due for screening and thus potentially eligible to be randomly selected for this study. Approximately 67% of screening-eligible patients at BRH are up-to-date with CRC screening, leaving about 33% (*n* = ~2,600) patients eligible to be selected for this screening study.

1.3 Purpose and Rationale

Although the effectiveness of mailed FIT-based screening programs has been demonstrated within organized health systems (Daly et al., 2010; Levy et al., 2012), it is unclear whether this approach is effective, feasible, acceptable, and cost-effective in community health centers, which tend to be under-resourced. The purpose of this study is to assess whether a mailed FIT outreach program can improve CRC screening completion among patients served by RCCHC and BRH. Using a randomized controlled trial study design, our study team will randomly assign patients to the following arms:

Arm 1 (control): Usual care

Arm 2 (intervention): Mailed FIT plus up to 2 reminder letters

We will compare the two arms on CRC screening completion rates. We will also assess and compare implementation outcomes for the intervention arm (Arm 2),

including whether the mailed FIT outreach program was feasible, acceptable, and cost-effective.

2.0 STUDY OBJECTIVES AND ENDPOINTS

2.1 Primary Objective (Aim 1)

The primary objective of this study is to assess the effectiveness of a mailed FIT outreach program on CRC screening completion in a community health center context in both northeastern and western North Carolina, by comparing the proportion of patients screened (as indicated by FIT or colonoscopy completion, assessed in the electronic health records [EHRs]), in the intervention arm and the control arm, 6 months post-intervention. Because both FIT and colonoscopy are viable CRC screening options and some patients may opt for a colonoscopy instead of the FIT, our measure of CRC screening completion includes both FIT and colonoscopy completion. We hypothesize that CRC screening completion rates will be higher among patients in Arm 2, compared to patients who received usual care in Arm 1.

2.2 Secondary Objectives

Other objectives of this study are to assess implementation determinants and outcomes to inform plans for sustaining and scaling the CRC screening program. Our aims are to:

Aim 2: Assess the logistical feasibility and patient and provider acceptability of the mailed FIT outreach program (Arm 2), and

Aim 3: Estimate the cost and cost-effectiveness per person screened in each arm, as well as the level of resource investment required to scale-up the program statewide.

3.0 PATIENT ELIGIBILITY

We will use the inclusion and exclusion criteria below to guide our electronic health record (EHR) query to identify eligible patients for this study.

3.1 Inclusion Criteria

Individuals must meet all of the following inclusion criteria to participate in this study:

3.1.1 Age 50-75 years

3.1.2 At average risk for CRC ("average risk" is defined as those patients who do not have any of the following: history of CRC, colonic adenomas, family history of CRC, or diagnosis of inflammatory bowel disease)

3.1.3 No record of fecal occult blood test (FOBT)/FIT within the past 12 months or colonoscopy within 10 years, sigmoidoscopy within 5 years, barium enema within 5 years, or computed tomography (CT) colonography within 10 years of the EHR query date

3.1.4 No record of any CRC diagnosis or total colectomy

3.1.5 Active patient (seen within the past 18 months) of RCCHC or BRH

Patients invited to participate in an interview will meet two additional inclusion criteria: be assigned to study Arm 2 and have a positive (abnormal) FIT result as a participant in the screening program.

3.2 Exclusion Criteria

All individuals meeting any of the exclusion criteria at baseline will be excluded from study participation:

3.2.1 Age younger than 50 years or older than 75 years

3.2.2 Not at average risk for CRC ("average risk" is defined as those patients who do not have any of the following: history of CRC, colonic adenomas, family history of CRC, or diagnosis of inflammatory bowel disease)

3.2.3 Record of FOBT/FIT within the past 12 months or colonoscopy within 10 years, sigmoidoscopy within 5 years; barium enema within 5 years, or CT colonography within 10 years of the EHR query date

3.2.4 Record of any CRC diagnosis or total colectomy

3.2.5 Not an active patient (not seen within the past 18 months) of RCCHC or BRH

Patients will not be invited to participate in an interview if they meet additional exclusion criteria: be assigned to study Arm 1, do not return a FIT as a participant in the screening program, or have a negative (normal) FIT result as a participant in the screening program.

4.0 STUDY PLAN

4.1 Schema

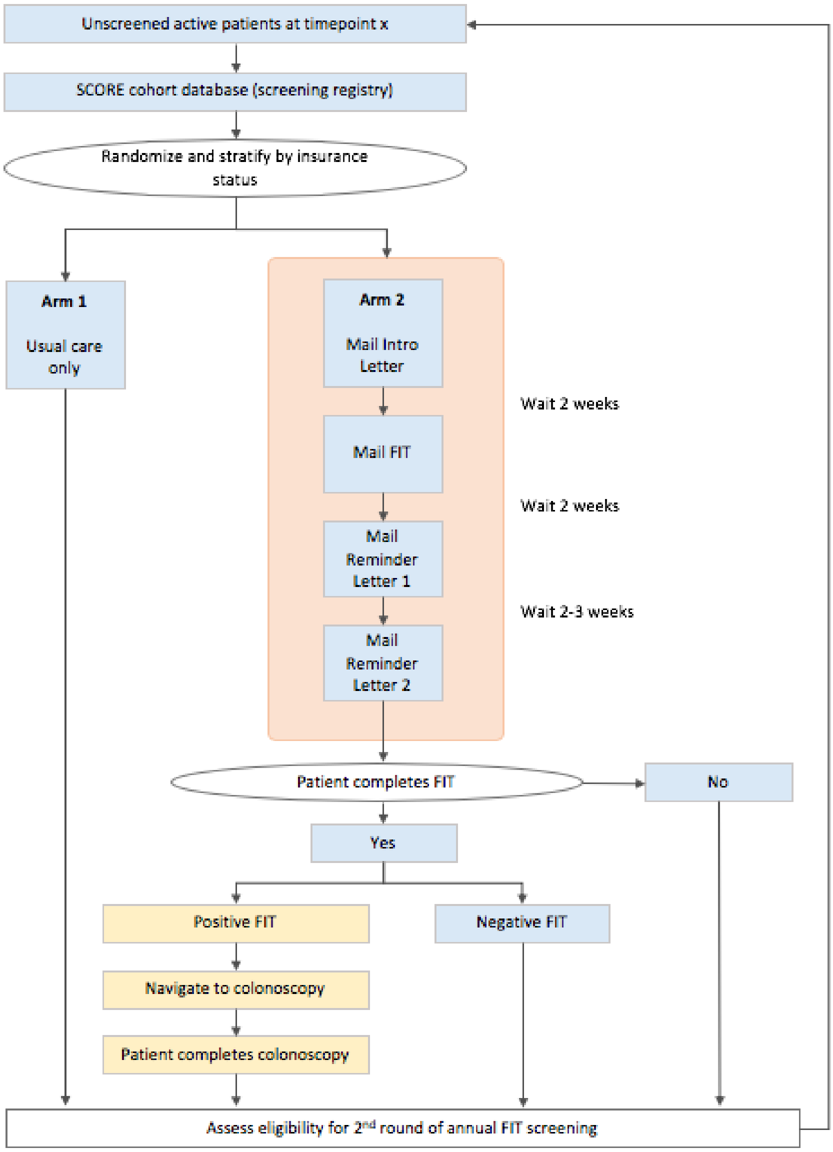

This trial will enroll up to 4,000 patients who are not up-to-date with CRC screening. The primary purpose of this study is to assess the effectiveness of mailed FIT outreach on CRC screening completion in a community health center context in both northeastern and western North Carolina, by comparing the proportion of patients screened across two study arms.

All participants assigned to Arm 2 will be eligible to receive two intervention rounds, scheduled about 54 weeks apart. These two intervention rounds will allow us to longitudinally assess adherence to the United States Preventive Services Task Force recommendations for annual screening with FIT (see 1.2). Arm assignment will remain the same across both intervention rounds. Patients will be

included in the second intervention round if they continue to meet the study's IRB-approved inclusion criteria for age, risk, screening history, CRC or colectomy history, and active patient status (seen within the past 18 months). In accordance with clinical recommendations, patients who receive a positive (abnormal) FIT result in the first round will not be included in the second round of FIT mailings (they will not be mailed a FIT).

We anticipate that the approximate time for the study team to complete each of the two rounds of study mailings will be 3 months, and patients' active participation during each round will last from 4 weeks (for patients with a FIT negative result and needed few, if any, reminders to complete the FIT) up to 6 months (for patients with a positive FIT result who need a follow-up colonoscopy, and needed several reminders to complete the FIT). For each round, we will assume that patients who have not completed screening (FIT, screening colonoscopy, or follow-up colonoscopy) after 6 months following the FIT kit mailing, do not intend to do so. We will randomly select an equal number of control group participants to correspond to each mailing wave for intervention participants, and will track both intervention and control participants for the same time period (from Introductory Letter mailing to 6-months post-mailing).

<u>Provider surveys:</u> All FQHC providers who deliver care to patients who received a mailed FIT (Arm 2) will be invited to complete a brief (about 18 items) survey to assess their perceptions of the appropriateness, feasibility, and fit of the program. We also will administer a brief (about 13 items) Contextual Determinants Survey to representatives of each FQHC to assess organizational climate, culture, and communication patterns that may affect the implementation of the project. The Contextual Determinants Survey will be administered to providers with leadership roles FQHC, such as the Chief Medical Officer, project champion, Clinic Nurse Managers, and Lead Referral Coordinators. Contextual Determinants Survey participants all have had direct contact with the UNC project team as we collaboratively developed the program.

<u>Provider interviews:</u> Approximately 2-3 months after administering the provider surveys, we will invite a cross-section of up to 20 providers to participate in a semi-structured interview. We will recruit providers who recommend or provide CRC screening to patients of each FQHC (10 providers from each FQHC region). The interviews will allow us to better understand contextual factors that may affect adoption, maintenance, and scalability of the SCORE CRC screening program.

<u>Patient interviews:</u> We will invite up to 30 intervention arm patients (up to 15 from each FQHC) to participate in a semi-structured interview to better understand factors that affect completion of a follow-up colonoscopy following a positive (abnormal) FIT result. A member of the project team will invite patients to participate in an interview at least 2 weeks after their final telephone contact with a patient navigator. We will sample purposively to ensure that we include

important and representative perspectives within each FQHC, based on sex, age, race, ethnicity, English/Spanish fluency, and whether the patient completed a follow-up colonoscopy.

4.2 Duration of Study

We anticipate that the entire active data collection phase, from randomizing the cohort to assessing screening outcomes via EHR and completing interviews with a subset of patients (see 4.3.6), will be approximately 36 months.

4.3 Study Details

4.3.1 Cohort Ascertainment

Our study team will work with RCCHC and BRH to identify the population of patients at each site who meet study inclusion criteria (see section 3.1), using an automated query of the EHR at each site. Per protocols from two similar projects, all patients meeting inclusion criteria will be initially included in the study cohort. Because EHR records are based on best available information and may not reflect screenings performed at another facility, our study materials will include a phone number for patients to contact the study team to self-identify as ineligible. We will select patients for inclusion randomly from the cohort and replace as needed to reach the goal of up to 4,000 from RCCHC and BRH. Each FQHC will query their own EHR to identify patients who meet eligibility criteria. Designated staff at each FQHC will securely transfer the queried patient information from their EHR to a folder accessible only to IRB-approved study team members on a secure UNC server (UNC Teams folder designated specifically for each FQHC). The data set will contain, but not necessarily be limited to, patient name, age, sex, race/ethnicity, email address, mailing address, primary care provider, and the date of the most recent visit with one of the FQHC's primary care providers. Only authorized clinic and UNC project team representatives will be able to upload or remove data to UNC Teams. Each FQHC site will have its own folder, which will prevent access to data of patients not seen at their clinic. A member of the UNC project team will then transfer the patient data from UNC Teams to a REDCap database accessible only to UNC project team members.

4.3.2 Randomization

At baseline, patients will be randomized 1:1 to Arm 1 (usual care) and Arm 2 (mailed FIT outreach), stratified by site (RCCHC, BRH) and insurance status (Medicare, Medicaid, commercial/private, self-pay/uninsured). The randomization list will be generated by the study biostatistician using a SAS program for stratified (by site and insurance status) randomization.

4.3.3 FIT Kit Packet Contents and Mailing

All FIT kit construction and mailing will be done by UNC project staff. For patients in Arm 2 (intervention arm), we will mail an introductory letter alerting patients to expect a free CRC screening test in the mail. Approximately two weeks later, we will mail a FIT Kit, which consists of a(n): 1) cover letter from

RCCHC or BRH reminding the patient s/he is due for CRC screening; 2) study information sheet; 3) education sheet about CRC screening; 4) instructions for completing the FIT; 5) one-sample FIT kit; and 6) pre-paid return mailer for the completed FIT kit (this return mailer includes a lab test form with the patient's name, address, date of birth, and medical record number). For pragmatic reasons, FIT kits will be mailed across multiple waves (rather than to all patients at once) over the course of several months. The FIT mailing materials were developed and tested during the pilot phase of this study. They have now been adopted by each clinic as their standard of care for mailed FIT outreach.

The mailing schedule is depicted in the schema (section 4.1). Patients in Arm 2 who do not return the FIT kit within 2-3 weeks, will receive Reminder Letter 1. If they do not return the FIT kit within 2-3 weeks after Reminder Letter 1, they will receive Reminder Letter 2.

4.3.4 FIT Return and Sample Processing

As described in section 4.3.3, participants in Arm 2 will be provided with a pre-paid return mailer for their completed FIT kit. This pre-paid mailer is addressed to the laboratory. Patients may mail the completed kit directly to the laboratory or can bring it to the FQHC, who will send the sample to the laboratory for processing. The laboratory will relay the test results to the appropriate FQHC. Each FQHC assigns appropriate FQHC staff members to ensure test results of all patients, regardless of study participation, are entered into the EHR per their usual protocols.

4.3.5 Notification of Results and Follow-up on Positive FITs

<u>Negative FIT result:</u> BRH patients with a negative FIT result, per BRH current clinical protocol, will receive a letter from their primary care provider. For RCCHC patients with a negative FIT result, IRB-approved UNC research staff will send patients a letter (approved by RCCHC) to notify them of the negative result. The letter will include a reminder that the test should be repeated in 12 months.

<u>Positive FIT result:</u> For patients with a positive (abnormal) FIT result, the patient's primary care provider or the provider's designee will call the patient, per current standard protocol at each FQHC. The primary care provider or primary care provider's designee will inform the patient of the positive result and need for a follow-up colonoscopy. After a patient is notified of a positive result, the patient navigator (employed by UNC and credentialed by each FQHC) will call the patient to offer information and support, including navigation services to access colonoscopy. Navigation services include, but are not necessarily limited to, referral and assistance with registering for charity care programs operating in each region (Hertford Health Access program for RCCHC and Digestive Health Partners-Hendersonville or Pardee Hospital Charity Care program for BRH) including no-cost colonoscopy (for uninsured patients) and, if available, transportation services to and from the colonoscopy appointment (for all patients).

Patients who do not qualify for charity care may receive no-cost colonoscopy, supported using study funding and/or donated colonoscopy services. The patient navigator will help navigate the patient to these services, if needed.

4.3.6 Data Collection

REDCap database: RCCHC and BRH will securely transfer patient information from the EHR to a secure SharePoint folder at UNC. A UNC research assistant will then upload the data to a REDCap database (developed based on two previous completed or ongoing studies). Data will include, but not necessarily be limited to, patient name, age, sex, race/ethnicity, mailing address, identified primary care provider, and if possible, the number of visits within the past 18 months with that provider. The study team has fully executed data use agreements in place with each FQHC. Designated approved members of the research team members will use the REDCap database to document whether patients were mailed a FIT kit and to track the status of FIT kit completion (yes or no), test result (positive or negative), contact with patients who have positive results (yes or no), and receipt of navigation support for colonoscopy (yes or no), including referrals for no-cost follow-up colonoscopy (yes or no) and transportation services (yes or no). Approved members of the UNC research team will also document in the REDCap database whether patients call to report they are ineligible for FIT screening per their screening and relevant medical history (e.g., already completed CRC screening elsewhere, have a medical history that places them at higher than average risk for CRC).

EHR: An RCCHC or BRH staff member will query the EHR 6 months after FIT packets are mailed to determine whether patients completed any CRC screening test (FIT, colonoscopy, flexible sigmoidoscopy, Cologuard) and, if so, the test result. As mentioned in the preceding paragraph, these data will be securely transferred to UNC and uploaded to the REDCap database for analysis.

Charity Care Program: Hertford Health Access will oversee no-cost follow-up colonoscopy and transportation service to and from medical appointments for RCCHC patients. Digestive Health Partners-Hendersonville and Pardee Hospital Charity Care program provides no-cost colonoscopy services for BRH patients. The patient navigator will contact the local charity care program (Hertford Health Access, Digestive Health Partners, or Pardee Hospital) to acquire data on whether patients from our cohort (who had a positive FIT result) received no-cost follow-up colonoscopy and transportation service. The patient navigator will store these data in the secure REDCap database.

Surveys and interviews: We will use brief surveys and semi-structured interviews to assess providers' perceptions about the acceptability, appropriateness, and feasibility of the mailed reminder plus FIT kit program. We also will recruit up to 15 patients from each FQHC (RCCHC and BRH) to participate in a 30-45-minute interview to better understand factors that affect their completion of a follow-up colonoscopy following a positive (abnormal) FIT result. Spanish-speaking patient

participants will be eligible to participate in the interviews. Patient interviews will be conducted by phone or in person. For provider interviews we will recruit up to 10 FQHC and GI providers associated with each FQHC site. Provider interviews will be conducted in person or by phone depending on the providers' preference. Interviews will be audio recorded and transcribed verbatim. Personally identifiable information will be redacted from the transcripts prior to analysis.

4.3.7 Number and Duration of Contact with Patients

4.3.7.1 CRC Screening

Patients in the control arm will not be contacted. For patients in the intervention arms, the study team will send an Introductory Letter (first contact) that notifies the patient to expect a FIT kit in the mail. This mailing will allow the study team to identify patients with incorrect addresses before mailing the FIT kit. If the mailing is returned to the study team because of an incorrect address, the patient will be removed from future mailings.

After mailing the FIT kit (second contact), the study team will make up to two reminder attempts to follow up with patients in the intervention arm (Arm 2) who have not returned a completed FIT kit. Patients in Arm 2 will receive up to two reminder letters (third and fourth contact).

<u>For patients who complete a FIT kit and have a negative result</u>: The patient will be notified of the test result by letter (see section 4.3.5).

<u>For patients who complete a FIT kit and have a positive (abnormal) result</u>: The patients' primary care provider or the primary care provider's designee will notify the patient of the results. After the patient is notified of their positive result, the patient navigator (employed by UNC and credentialed by RCCHC and BRH) will contact the patient by phone or, if phone contact is not successful, by mail to offer information and support for accessing follow-up colonoscopy. Communication will include information about no-cost colonoscopy (for uninsured patients) and transportation service to and from the colonoscopy appointment (for all patients). The patient navigator will call the patient up to three additional times to support the patient in scheduling an appointment for a follow-up colonoscopy, preparing for the colonoscopy procedure, accessing transportation, and financial services, and understanding colonoscopy results. The approximate duration of each phone call will be 5-15 minutes.

The total number of contacts initiated by the study team will range from **3** (Introductory Letter + FIT kit mailing + results letter/call) to **9** (Introductory Letter + FIT kit mailing + up to 2 reminder letters + results letter/call + up to 4 navigation calls for patients with a positive FIT). The study team may have additional contact with patients if the patients reach out to the study team or

indicate they require additional contact (e.g., questions or requests for navigation assistance).

4.3.7.2 Provider Surveys

Survey completion will entail a minimum of 2 contacts at each of the 2 time points. The first contact will be a recruitment email and the second contact will be an online survey. Additional contacts may include up to 4 recruitment emails and a brief presentation during a standing provider meeting, to invite participation. The brief survey will require 10 minutes or less.

4.3.7.3 Provider Interviews

Interview completion will entail a minimum of 4 contacts at each of 2 time points. The first contact will be a recruitment email, the second contact will an interview scheduling call or email, the third contact will be an interview reminder, and the fourth contact will be an interview. Additional contacts may include up to 6 recruitment emails and/or phone calls and a brief presentation during a standing provider meeting, to invite participation. The interview will require approximately 20 minutes.

4.3.7.4 Patient Interviews

We expect at least 5 contacts including a recruitment letter, a recruitment phone call to schedule the interview, an interview reminder, the interview, and a post-interview contact to mail the gift card. We also will call patients up to 5 times for interview recruitment. Interviews will last approximately 30-45 minutes.

4.4 Expected Risks

It is possible that patients may experience emotional distress or feelings of embarrassment of completing a home-based stool test. However, given that CRC screening using FIT is a part of usual care at the participating FQHCs, these risks are not substantially increased as a result of study participation. The letters included with the mailed FIT intervention emphasize the benefits of the screening, that participation is voluntary, they may choose not to answer questions, there will be no effects on care received by the FQHC or providers based on decision about participation, and information collected will be confidential and securely stored.

4.5 Removal of Patients from Protocol

We have not identified any factors *a priori* that would lead the Principal Investigator to remove a patient from the study.

5.0 TIME AND EVENTS TABLE

5.1 Time and Events Table

The timeline of activities for our study is shown in Table 1.

Table 1. Project timeline

Activity	Year and Quarter (Q)											
	Year 1				Year 2				Year 3			
	Q1	Q2	Q3	Q4	Q1	Q2	Q3	Q4	Q1	Q2	Q3	Q4
**Round 1**												
Identify eligible patients (query EHR)	■	■	■	■								
Transfer patient data to UNC and REDCap	■	■	■	■								
Implement mailed FIT outreach (in waves)	■	■	■	■								
FIT tracking and other data collection	■	■	■	■	■	■						
FIT results notification	■	■	■	■	■	■						
Patient navigation for patients with a positive (abnormal) FIT result	■	■	■	■	■	■						
Provider surveys		■										
Provider interviews			■	■								
Patient interviews		■	■	■	■							
**Round 2**												
Identify eligible patients (query EHR)					■	■	■	■				
Transfer patient data to UNC and REDCap					■	■	■	■				
Implement mailed FIT outreach (in waves)					■	■	■	■				
FIT tracking and other data collection						■	■	■	■	■		
FIT results notification						■	■	■	■	■		
Patient navigation for patients with a positive (abnormal) FIT result						■	■	■	■	■		
Provider surveys							■					
Provider interviews								■				
Patient interviews						■	■	■				
**Outcomes assessment and evaluation**												
CRC screening outcomes assessment			■	■	■	■	■	■	■	■		
Cost and cost-effectiveness assessment	■	■	■	■	■	■	■	■	■	■	■	■
Program evaluation	■	■	■	■	■	■	■	■	■	■	■	■

6.0 UNANTICIPATED PROBLEMS

6.1 Definition

As defined by UNC's IRB, unanticipated problems involving risks to study subjects or others (UPIRSO) refers to any incident, experience, or outcome that:

- Is unexpected (in terms of nature, severity, or frequency) given (a) the research procedures that are described in the protocol-related documents, such as the IRB-approved research protocol and informed consent document; and (b) the characteristics of the subject population being studied;
- Is related or possibly related to a subject's participation in the research; and
- Suggests that the research places subjects or others at a greater risk of harm (including physical, psychological, economic, or social harm) related to the research than was previously known or recognized.

6.2 Reporting

Any UPIRSO that occurs during the conduct of this study and that meets all three criteria listed in 6.1 must be reported to the UNC IRB using the IRB's web-based reporting system.

7.0 STATISTICAL CONSIDERATIONS

7.1 Study Design

This study includes a two-arm, multi-center, randomized controlled trial. The primary objective of the trial is to assess the effectiveness of a mailed FIT outreach program on CRC screening completion in a community health center context in both northeastern and western North Carolina, by comparing the proportion of patients screened in the intervention arm to the control arm. Arm 1. Participants receive usual care (no change from the original study protocol). Arm 2. Participants receive the following already IRB-approved intervention components: (1) Introductory Letter to let patients know they should expect a FIT kit in the mail; (2) Cover Letter and FIT kit with instructions; (3) Reminder Letter 1 for patients who have not returned the FIT kit; and (4) Reminder Letter 2 for patients who still have not returned the FIT kit (added in protocol version 2).

The secondary objective of this study is to develop and refine implementation outcomes to inform a large, multi-site implementation study. As part of this secondary objective, our aims are to test the logistical feasibility and patient and provider acceptability of a mailed reminder plus FIT outreach program (Aim 2), and estimate the cost per person screened of the mailed reminder plus FIT outreach program, as well as the level of resource investment required to scale-up the program statewide (Aim 3).

7.2 Sample Size and Accrual

Our project funding supports a sample size of up to 4,000 patients at two clinical sites who are eligible for CRC screening. Using a 1:1 randomization approach, up to 2,000 RCCHC patients and up to 2,000 BRH patients will be randomized to the two study arms. For each FQHC, up to 1,000 patients will be randomized to each of the two arms. Our statistician performed formal power calculations for our primary study outcome comparing CRC screening completion for the intervention arm vs. the control arm, stratified by patient insurance status (4 strata: Medicare, Medicaid, commercial/private, and self-pay/other). Based on previous research, we anticipate that 25% of those receiving the intervention will complete CRC screening, and 17% of patients in the usual care arm will complete screening (8% difference between arms). We need 492 participants per arm, per strata to have at least 80% power to detect an 8% difference between study arms at 1-sided alpha level 0.0125 (3,936 participants total, which can be rounded up to 4,000). We calculated power by SAS PROC Power (SAS Institute, Cary, NC).

7.3 Data Analysis Plans

Our main statistical test for primary and secondary outcomes will be a Mantel-Haenszel chi-squared test, adjusted for recruitment site (FQHC). If there are important differences between the control and intervention arms across baseline variables known to be associated with the outcome being tested, we will also then use multiple logistic regression, adjusting for the additional baseline variables. One-sided tests will be used for the primary and secondary outcomes because we expect active interventions will lead to more favorable results compared to usual care.

7.3.1 Aim 1

For Aim 1, the main outcome of interest is the proportion of the sample completing FIT kits among those to whom the materials are mailed. We refer to this metric as the "proportion screened." Using a 1:1 randomization approach, up to 2,000 RCCHC patients and up to 2,000 BRH patients will be randomized to each study arm (Arm 1-usual care; Arm 2-introductory letter + mailed FIT + 2 follow-up reminder letters).

Aim 1 analytic approach: Our primary analysis will be a crude comparison of proportions of completed CRC screening at 6 months post-FIT mailing for the mailed FIT plus reminder arm vs. the usual care arm using a one-sided Mantel-Haenszel chi-squared test, controlling for recruitment site (FQHC) and insurance status. As stated in the overview, we will also, if necessary, conduct a multiple logistic regression analysis. We will also compare the proportion screened between the mailed FIT plus reminder arm vs. the usual care arm for each of the four insurance status groups, using a one-sided Mantel-Haenszel chi-squared test, controlling for recruitment site, conducted at the 0.0125 significance level.

7.3.2 Aim 2

For Aim 2, we will examine intervention implementation outcomes, including the logistical feasibility, patient acceptability, and provider acceptability of a mailed FIT outreach program. To accomplish this aim, we will track and report feasibility and acceptability metrics. For feasibility, we will measure the proportion of bad addresses using the REDCap database. To assess patient acceptability and perceptions of program appropriateness, we will conduct semi-structured interviews with up to 30 patients who had a positive (abnormal) FIT result, were referred for follow-up colonoscopy, and were offered patient navigations services. Invitations will be made on a rolling basis until a sufficient number of patients complete the interviews. Invitation letters will be sent after no more than 6 months after the FIT kit was mailed. To assess acceptability and feasibility from the providers' perspectives, we will conduct surveys and semi-structured interviews at two time points. For the interviews, the RCCHC or BRH medical director will send an email to the clinician listserv to invite clinicians at the target clinic to participate in a follow-up interview. If this approach does not yield enough clinician participants, UNC project staff will attend regularly scheduled

provider meetings to enhance recruitment. Clinician interviews will be conducted in study months 6-12. We will also track and report the proportions of patients who inform us of ineligibility in response to the Introductory Letter and subsequent mailings.

Aim 2 analytic approach: For quantitative assessments, we will use descriptive statistics (e.g., proportions and associated 95% confidence intervals) to characterize feasibility and acceptability measures. For qualitative assessments (i.e., semi-structured interviews), we will perform a thematic analysis of transcribed interview data.

7.3.3 Aim 3

For Aim 3, the main outcome of interest is the total cost (sum of fixed and variable costs) and the incremental cost-effectiveness of the intervention arm versus the usual care arm, measured in cost per person screened for CRC. We will document all fixed and variable costs associated with implementing the intervention, based upon personnel time tracking, invoices, receipts paid, test kit and processing costs, postage and other mailing costs, and other means. Fixed costs (i.e., costs absorbed by the screening program irrespective of the number of participants screened) include those of program administration and setup, materials design, and database development and analytics. Variable costs (i.e., those that are determined by the number of participants engaged or screened) include the costs of the intervention materials, FIT kits, postage and mailing costs, and costs of patient navigation and other personnel involved in the screening and follow-up process. For personnel time (e.g., in-reach follow-up of non-responders; navigation to colonoscopy), we will record the amount of time in minutes spent on each participant interaction, and calculate cost by multiplying total time spent in minutes with the average hourly salary of participating clinic nurses, administrative staff, and other staff, as relevant. Time spent in training, handling samples, and data management will be monitored using a similar time-audit approach. Mailing-associated costs will be recorded for the intervention arm. We will also assess costs associated with additional health care utilization post-screening (e.g., follow-up colonoscopy). We will not assess fixed costs common across the arms, such as overhead operating costs of clinics, or costs related to the conduct of the study alone, such as costs associated with data collection. For secondary analyses using the societal perspective, we will also attempt to estimate patient time costs associated with the intervention arm versus usual care arm.

Aim 3 analytic approach: We will describe total costs associated with each of the intervention arms for the trial. We will then calculate the incremental cost-effectiveness ratio (ICER) defined as cost per additional person screened, comparing intervention arms to usual care. We will also perform sensitivity analyses to examine the effect of different prices (potentially negotiable for larger programs assuming volume-based economies of scale). For alternate analyses

from the societal perspective, we will estimate patient time costs using the human capital method (Jonas et al., 2008).

7.4 Data Management/Audit

Study team members will enter data into a REDCap database. Study data will be stored securely and managed through the REDCap database.

As an investigator initiated study, this trial will also be audited by the Lineberger Cancer Center audit committee every six or twelve months, depending on the participation of affiliate sites.

8.0 STUDY MANAGEMENT

8.1 Institutional Review Board (IRB) Approval and Consent

It is expected that the IRB will have the proper representation and function in accordance with federally mandated regulations. The IRB should approve the consent form and protocol.

In obtaining and documenting informed consent, the investigator should comply with the applicable regulatory requirement(s), and should adhere to Good Clinical Practice (GCP) and to ethical principles that have their origin in the Declaration of Helsinki.

For mailed outreach participants: We will request a waiver of informed consent for mailed outreach participants because: 1) the research involves no more than minimal risk to the subjects; 2) the waiver will not adversely affect the rights and welfare of the subjects; and 3) the research could not practicably be carried out without the waiver.

For patient interview participants: Before recruitment and enrollment onto this study, the patient will be given a full explanation of the study and will be given the opportunity to review the consent form. Each consent form must include all the relevant elements currently required by the FDA Regulations and local or state regulations. Once this essential information has been provided to the patient and the investigator is assured that the patient understands the implications of participating in the study, the patient will be asked to give consent to participate in the study by signing an IRB-approved consent form.

Prior to a patient's participation in the trial, the written informed consent form should be signed and personally dated by the patient and by the person who conducted the informed consent discussion.

For provider survey participants: The online survey will include a study information page. Providers will indicate consent by proceeding to the online survey after reviewing the study information page. The brief survey asks providers to share their professional opinions about the mailed FIT outreach

program and implementation of CRC screening at their clinic. The questions do not request sensitive information.

For provider interview participants: We will request verbal consent only because we are inquiring about participants' jobs, not personal information about themselves.

8.2 Required Documentation

Before the study can be initiated at any site, the following documentation must be provided to the Clinical Protocol Office (CPO) at the University of North Carolina.

- A copy of the official IRB approval letter for the protocol and informed consent
- CVs and medical licensure for the principal investigator and any associate investigators who will be involved in the study
- A copy of the IRB-approved consent form

8.3 Registration Procedures

Please describe how you will keep track of patient enrollment.

8.4 Adherence to the Protocol

Except for an emergency situation in which proper care for the protection, safety, and well-being of the study patient requires alternative treatment, the study shall be conducted exactly as described in the approved protocol.

8.4.1 Emergency Modifications

UNC investigators may implement a deviation from, or a change of, the protocol to eliminate an immediate hazard(s) to trial subjects without prior UNC IRB approval.

For any such emergency modification implemented, a UNC IRB modification form must be completed by UNC Research Personnel within five (5) business days of making the change.

8.4.2 Single Patient/Subject Exceptions

Eligibility single subject exceptions are not permitted for Lineberger Comprehensive Cancer Center Investigator Initiated Trials under any circumstances. Other types of single subject exceptions may be allowed if proper regulatory review has been completed in accordance with Lineberger Comprehensive Cancer Center's Single Subject Exceptions Policy.

8.4.3 Other Protocol Deviations/Violations

According to UNC's IRB, a protocol deviation is any unplanned variance from an IRB approved protocol that:

- Is generally noted or recognized after it occurs

- Has no substantive effect on the risks to research participants
- Has no substantive effect on the scientific integrity of the research plan or the value of the data collected
- Did not result from willful or knowing misconduct on the part of the investigator(s).

An unplanned protocol variance is considered a violation if the variance meets any of the following criteria:

- Has harmed or increased the risk of harm to one or more research participants.
- Has damaged the scientific integrity of the data collected for the study.
- Results from willful or knowing misconduct on the part of the investigator(s).
- Demonstrates serious or continuing noncompliance with federal regulations, State laws, or University policies.

If a deviation or violation occurs please follow the guidelines below:

Protocol Deviations: UNC personnel will record the deviation in OnCore® (or other appropriate database set up for the study), and report to any sponsor or data and safety monitoring committee in accordance with their policies. Deviations should be summarized and reported to the IRB at the time of continuing review.

Protocol Violations: Violations should be reported by UNC personnel within one (1) week of the investigator becoming aware of the event using the same IRB online mechanism used to report UPIRSO.

Unanticipated Problems Involving Risks to Subjects or Others (UPIRSO: Any events that meet the criteria for “Unanticipated Problems” as defined by UNC’s IRB (see section 6.1) must be reported by the Study Coordinator using the IRB’s web-based reporting system.

8.5 Amendments to the Protocol

Should amendments to the protocol be required, the amendments will be originated and documented by the Principal Investigator at UNC. It should also be noted that when an amendment to the protocol substantially alters the study design or the potential risk to the patient, a revised consent form might be required.

The written amendment, and if required the amended consent form, must be sent to UNC’s IRB for approval prior to implementation.

8.6 Record Retention

Study documentation includes all Case Report Forms, data correction forms or queries, source documents, Sponsor-Investigator correspondence, monitoring

logs/letters, and regulatory documents (e.g., protocol and amendments, IRB correspondence and approval, signed patient consent forms).

Source documents include all recordings of observations or notations of clinical activities and all reports and records necessary for the evaluation and reconstruction of the clinical research study.

Government agency regulations and directives require that all study documentation pertaining to the conduct of a clinical trial must be retained by the study investigator. In the case of a study with a drug seeking regulatory approval and marketing, these documents shall be retained for at least two years after the last approval of marketing application in an International Conference on Harmonization (ICH) region. In all other cases, study documents should be kept on file until three years after the completion and final study report of this investigational study.

8.7 Obligations of Investigators

The Principal Investigator is responsible for the conduct of the clinical trial at the site in accordance with Title 21 of the Code of Federal Regulations and/or the Declaration of Helsinki. The Principal Investigator is responsible for personally overseeing the treatment of all study patients. The Principal Investigator must assure that all study site personnel, including sub-investigators and other study staff members, adhere to the study protocol and all FDA/GCP/NCI regulations and guidelines regarding clinical trials both during and after study completion.

The Principal Investigator at each institution or site will be responsible for assuring that all the required data will be collected and entered onto the Case Report Forms. Periodically, monitoring visits will be conducted and the Principal Investigator will provide access to his/her original records to permit verification of proper entry of data. At the completion of the study, all case report forms will be reviewed by the Principal Investigator and will require his/her final signature to verify the accuracy of the data.

9.0 REFERENCES

Centers for Disease Control and Prevention. Vital signs: colorectal cancer screening test use—United States, 2012. MMWR Morb Mortal Wkly Rep. 2013;62(44):881-888.

Church TR, Yeazel MW, Jones RM, et al. A randomized trial of direct mailing of fecal occult blood tests to increase colorectal cancer screening. 2004; 96(10):770-780.

Daly JM, Levy BT, Merchant ML, Wilbur J. Mailed fecal-immunochemical test for colon cancer screening. J Community Health. 2010;35(3):235-239.

Gupta S, Halm EA, Rockey DC, et al. Comparative effectiveness of fecal immunochemical test outreach, colonoscopy outreach, and usual care for boosting colorectal cancer screening among the underserved: A randomized clinical trial. JAMA Intern Med. 2013;173(18):1725-1732.

Jonas DE, Russell LB, Sandler RS, Chou J, Pignone MP. Value of patient time invested in the colonoscopy screening process: Time requirements for colonoscopy study. Med Decis Making. 2008;28(1):56-65.

Kempe KL, Shetterly SM, France EK, Levin TR. Automated phone and mail population outreach to promote colorectal cancer screening. Am J Manag Care. 2012;18(7):370-378.

Levy BT, Daly JM, Xu Y, Ely JW. Mailed fecal immunochemical tests plus educational materials to improve colon cancer screening rates in Iowa Research Network (IRENE) practices. J Am Board Fam Med. 2012;25(1):73-82.

National Colorectal Cancer Roundtable. Working toward the shared goal of 80% screened for colorectal cancer by 2018. n.d.; http://nccrt.org/what-we-do/80-percent-by-2018/. Accessed May 1, 2018.

Office of Disease Prevention and Health Promotion. Healthy People 2020. 2014; https://www.healthypeople.gov/2020/topics-objectives/topic/cancer/objectives. Accessed May 2, 2018.

Sabatino SA, Lawrence B, Elder R, et al. Effectiveness of interventions to increase screening for breast, cervical, and colorectal cancers: Nine updated systematic reviews for the guide to community preventive services. Am J Prev Med. 2012;43(1):97-118.

Siegel RL, Sahar L, Robbins A, Jemal A. Where can colorectal cancer screening interventions have the most impact? Cancer Epidemiol Biomarkers Prev. 2015;24(8):1151-1156.

U.S. Cancer Statistics Working Group. United States Cancer Statistics: 1999–2010 Incidence and Mortality Web-based Report. 2013; http://www.cdc.gov/uscs. Accessed April 16, 2014.

U.S. Preventive Services Task Force, Bibbins-Domingo K, Grossman DC, et al. Screening for colorectal cancer: US Preventive Services Task Force recommendation statement. JAMA. 2016; 315(23):2564-2575.
