# Supplementary figures and images for: Time to Follow-Up Colonoscopy After Positive Fecal Immunochemical Test with Centralized Patient Navigation: A Randomized Clinical Trial
Source: J Gen Intern Med. 2026 Feb 4;41(6):1534–44. doi: 10.1007/s11606-025-10151-2 (PMC13007977; doi:10.1007/s11606-025-10151-2)

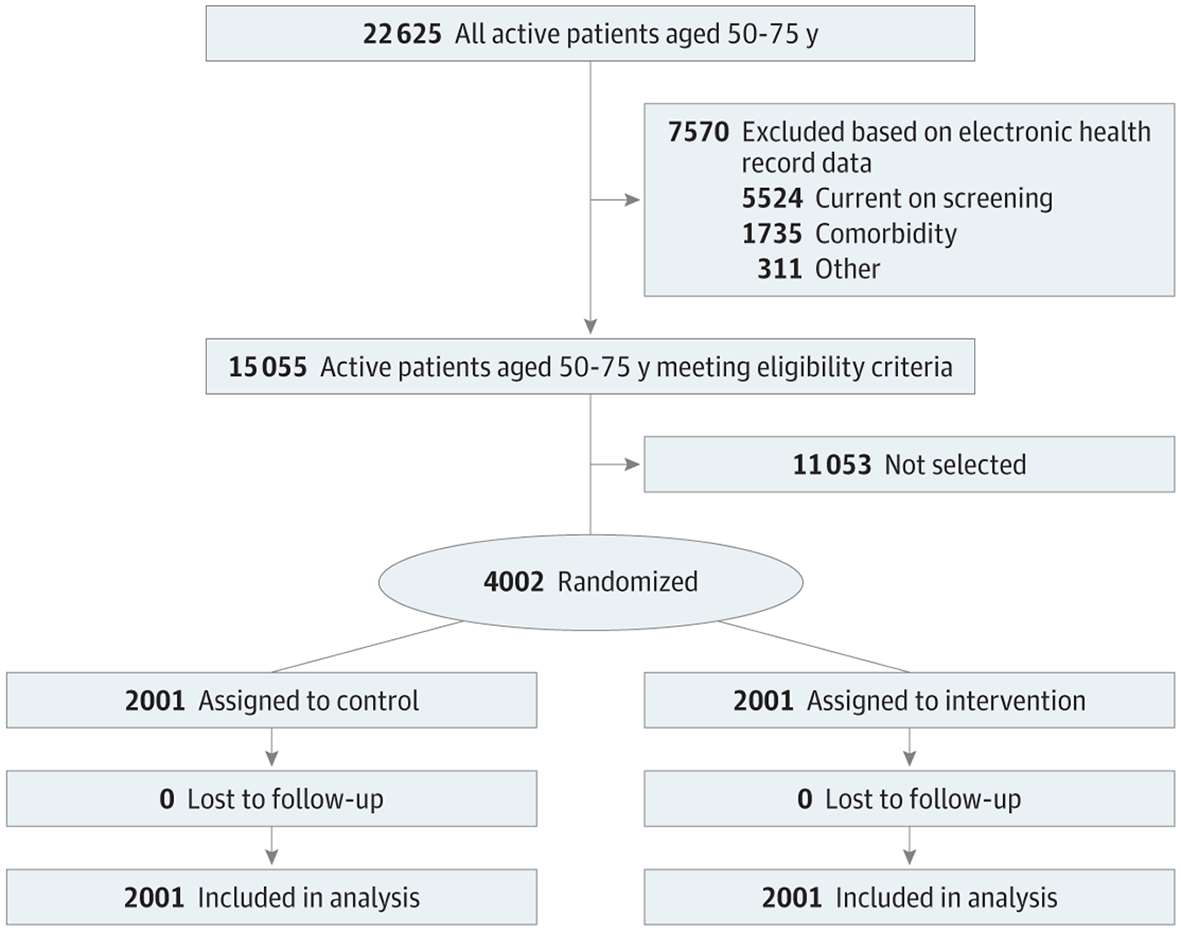

Supplement: Supplementary file 3 — (PNG 205 KB) [file 11606_2025_10151_Fig6_ESM.png]

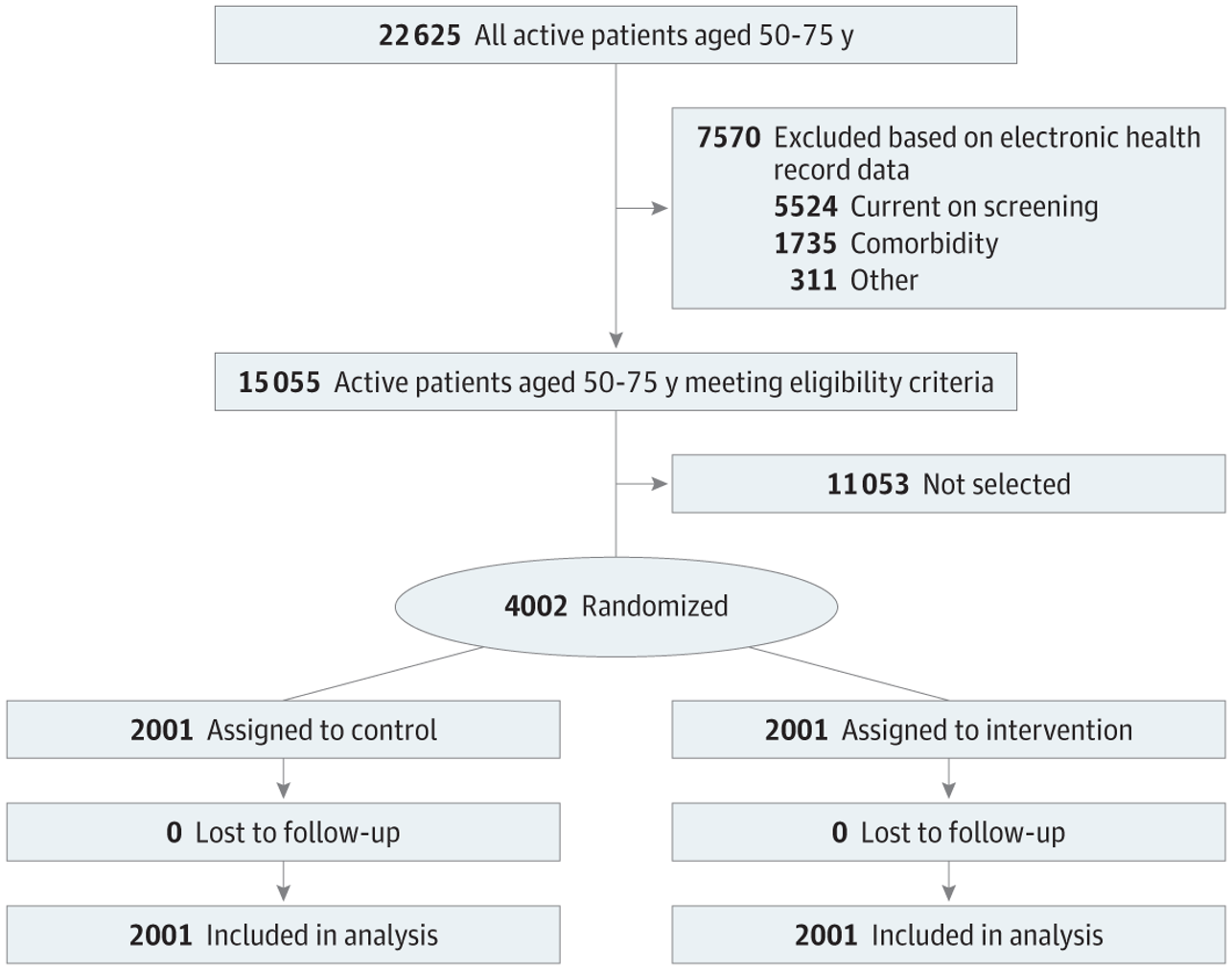

Supplement: Supplementary file 4 — (TIF 409 KB) [file 11606_2025_10151_MOESM3_ESM.tif]
